# A dataset of lung ultrasound images for automated AI-based lung disease classification

**DOI:** 10.1016/j.dib.2025.112034

**Published:** 2025-09-06

**Authors:** Andrew Katumba, Sudi Murindanyi, Nixson Okila, Joyce Nakatumba-Nabende, Cosmas Mwikirize, Jonathan Serugunda, Samuel Bugeza, Anthony Oriekot, Juliet Bossa, Eva Nabawanuka

**Affiliations:** a Department of Computer Science, Makerere University, Kampala, Uganda; b Department of Electrical and Computer Engineering, Makerere University, Kampala, Uganda; c Department of Radiology Makerere University Hospital, Makerere University, Kampala, Uganda; d Makerere Center for Artificial Intelligence and Data Science, Makerere University, Kampala, Uganda; e Mulago Specialised Women and Neonatal Hospital, Kampala, Uganda; f Mulago National Referral Hospital, Kampala, Uganda; g Emergent AI, Kampala, Uganda

**Keywords:** Lung ultrasound, Pulmonary disease, Deep learning

## Abstract

Lung ultrasound (LUS) is increasingly recognized as a valuable imaging modality for evaluating various pulmonary conditions. Despite its clinical utility, accurate interpretation of LUS remains challenging due to factors such as inter-operator variability, dependence on sonographer expertise, and inherently low signal-to-noise ratios. This article presents a curated benchmark dataset of labelled LUS images acquired in Uganda, intended to support the development of automated, AI-based diagnostic tools for lung disease classification. The dataset comprises 1062 labelled images collected from patients at Mulago National Referral Hospital and Kiruddu Referral Hospital by senior radiologists. The dataset is suitable for training and evaluating convolutional neural network-based models and is expected to facilitate research in developing robust deep learning systems for pulmonary disease diagnosis using LUS.

Specifications Table

**Table:** 

Subject	Applied Artificial Intelligence, Medical Imaging, and Computer Vision in Healthcare
Specific subject area	Deep learning for automated classification of lung diseases in lung ultrasound images
Type of data	Raw, Filtered, Processed Images, text
Data collection	Radiologists collected the data using Clarius C3 and C5 ultrasound probes. Ultrasound scans were conducted both longitudinally and transversely across 12 thoracic regions per patient, resulting in 24 images per patient.
Data source location	The data was collected from Mulago and Kiruddu National Referral Hospitals in Kampala, Uganda
Data accessibility	Repository name: Mendeley Data Data identification number: 10.17632/hb3p34ytvx.1 Direct URL to original unprocessed and processed data: https://data.mendeley.com/datasets/hb3p34ytvx/2 Direct URL to code for sorting, splitting, and processing data: https://github.com/Marconi-Lab/lung-ultrasound-ai-disease-classification
Related research article	Okila N, Katumba A, Nakatumba-Nabende J, Mwikirize C, Murindanyi S, Serugunda J, Bugeza S, Oriekot A, Bossa J and Nabawanuka E (2025) Deep learning for accurate B-line detection and localization in lung ultrasound imaging. Front. Artif. Intell. 8:1560,523. doi: 10.3389/frai.2025.1560523

## 1 Value of the Data

• The dataset comprises LUS images covering three categories of lung conditions and four classes of artifacts/features essential for advancing AI-driven LUS analysis. This supports early screening and monitoring of lung diseases, particularly in underserved populations.
• This dataset is a valuable resource for developing AI-assisted educational tools to train medical professionals in lung ultrasound interpretation.
• Potential for benchmarking and validation. Other researchers can use this dataset to benchmark their deep learning algorithms against existing models. To facilitate consistent and meaningful comparison of algorithm performance across studies, the use of evaluation metrics such as accuracy, sensitivity, specificity, and F1-score is recommended.
• Facilitating cross-disciplinary research. The dataset facilitates interdisciplinary research by fostering collaborations among AI researchers, radiologists, and clinicians. It supports studies in medical image processing, driving innovations in LUS image analysis. Furthermore, it contributes to developing explainable AI methods for clinical decision support systems.
• The dataset has been piloted in a study involving state-of-the art convolutional neural networks (CNNs) and a custom-designed CNN architecture for the automatic classification of lung conditions. Key research questions explored included: *To what extent can AI models accurately distinguish COVID-19 from other pulmonary pathologies using LUS images? And Can explainable AI approaches such as Grad-CAM, effectively identify clinically relevant regions in LUS images for validation by radiologists?*
• The dataset comprises a total of 149 patients, with ages ranging from 19 to 93 years and a mean age of approximately 40.85 years. The gender distribution includes 111 males (74.5 %) and 38 females (25.5 %). In terms of comorbidities, 27 patients (18.1 %) were classified as “Probably Covid,” 67 patients (45.0 %) had a “Diseased lung but probably not Covid,” and 55 patients (36.9 %) were noted to have a “Healthy Lung.

## 2 Background

Lung diseases represent a significant global health burden, contributing substantially to morbidity and mortality worldwide [1]. Timely and accurate diagnosis is critical for reducing disease-related fatalities and improving clinical outcomes. Lung ultrasound (LUS) has gained prominence in recent years as a non-invasive, radiation-free imaging modality for assessing a range of pulmonary conditions. However, its diagnostic utility is limited by operator dependency and specialised training requirements, posing particular challenges in low-resource settings where experienced personnel are scarce. These barriers constrain the widespread use of LUS outside specialized or acute care environments.

To address these limitations, recent research efforts [[2], [3], [4], [5], [6]] have investigated the use of deep learning techniques to automate LUS interpretation, showing promising results in enhancing diagnostic accuracy. Nonetheless, many of these models have been trained on datasets that do not reflect African populations' demographic and clinical characteristics, thereby limiting their generalizability in such contexts. The dataset presented in this article comprises a curated collection of LUS images from Ugandan healthcare settings, representing a previously underrepresented population in this domain. It aims to support the development and validation of more generalizable deep learning models for automated classification of pulmonary diseases.

## 3 Data Description

**Fig. 1:**
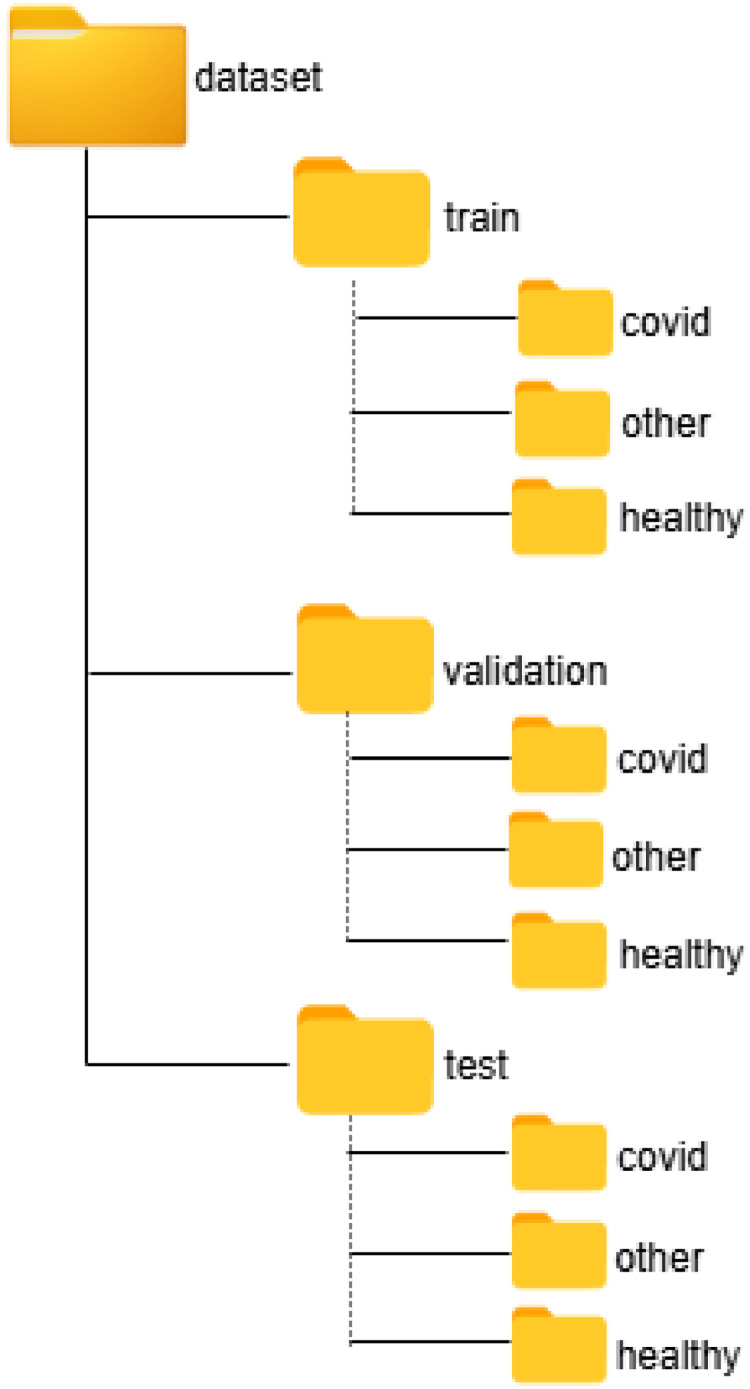
Directory structure of the lung ultrasound dataset. The dataset is organized into three main subsets: train, validation, and test, each containing class-specific subfolders for covid, other (Diseased Lung but Not COVID-19), and healthy images.

The dataset consists of lung ultrasound images categorized into three diagnostic classes: *Probably COVID-19* (COVID-19), *Diseased Lung but Probably Not COVID-19* (Other Lung Disease), and *Healthy Lung*. The data are partitioned into three primary subsets: train, validation, and test, by splitting at the patient level using folder identifiers. This approach ensures that images from the same patient are assigned exclusively to a single subset, effectively preventing data leakage. Within each primary folder, the images are further organized into three class-specific subfolders: covid, other, and healthy as illustrated in the Fig. 1 below.

Each image in the class-specific subfolders is saved in JPEG or PNG format, with resolutions ranging from 286 × 331 to 513 × 665 pixels. The image file naming followed two conventions: (i) anatomically descriptive names indicating scan location, orientation, and timestamp, and (ii) system-generated names composed of a unique identifier, frame index, and time of capture. The distribution of images across subsets and classes is summarized in Table 1.

**Table 1:** Distribution of lung ultrasound images across the dataset partitions (*train, validation*, and *test*).

Main folder	Sub-folder			Total
	covid	other	healthy	
train	241	241	241	723
validation	66	66	66	198
test	31	55	55	141

To support secondary use of the dataset, Table 2 provides sample metadata fields associated with the lung ultrasound data. This sample includes six patient entries, each annotated with key demographic and diagnostic information. The metadata fields include Patient Number, Age, Gender, and Conclusion (clinical diagnosis based on lung ultrasound), covering the three diagnostic categories: Healthy Lung, Probably COVID-19, and Diseased Lung but Probably Not COVID-19.

**Table 2:** Sample metadata showing key patient fields associated with the lung ultrasound images, including Patient Number, Age, Gender, and Clinical Conclusion.

Patient Number	Age	Gender	Conclusion
COAST-053	20	Male	Healthy Lung
COAST-017	50	Male	Probably Covid
COAST 061	63	Male	Diseased lung but probably Not Covid
COAST 062	28	Female	Diseased lung but probably Not Covid
COAST 125	52	Female	Healthy Lung
COAST 156	25	Female	Probably Covid

## 4 Experimental Design, Materials and Methods

**Fig. 2:**
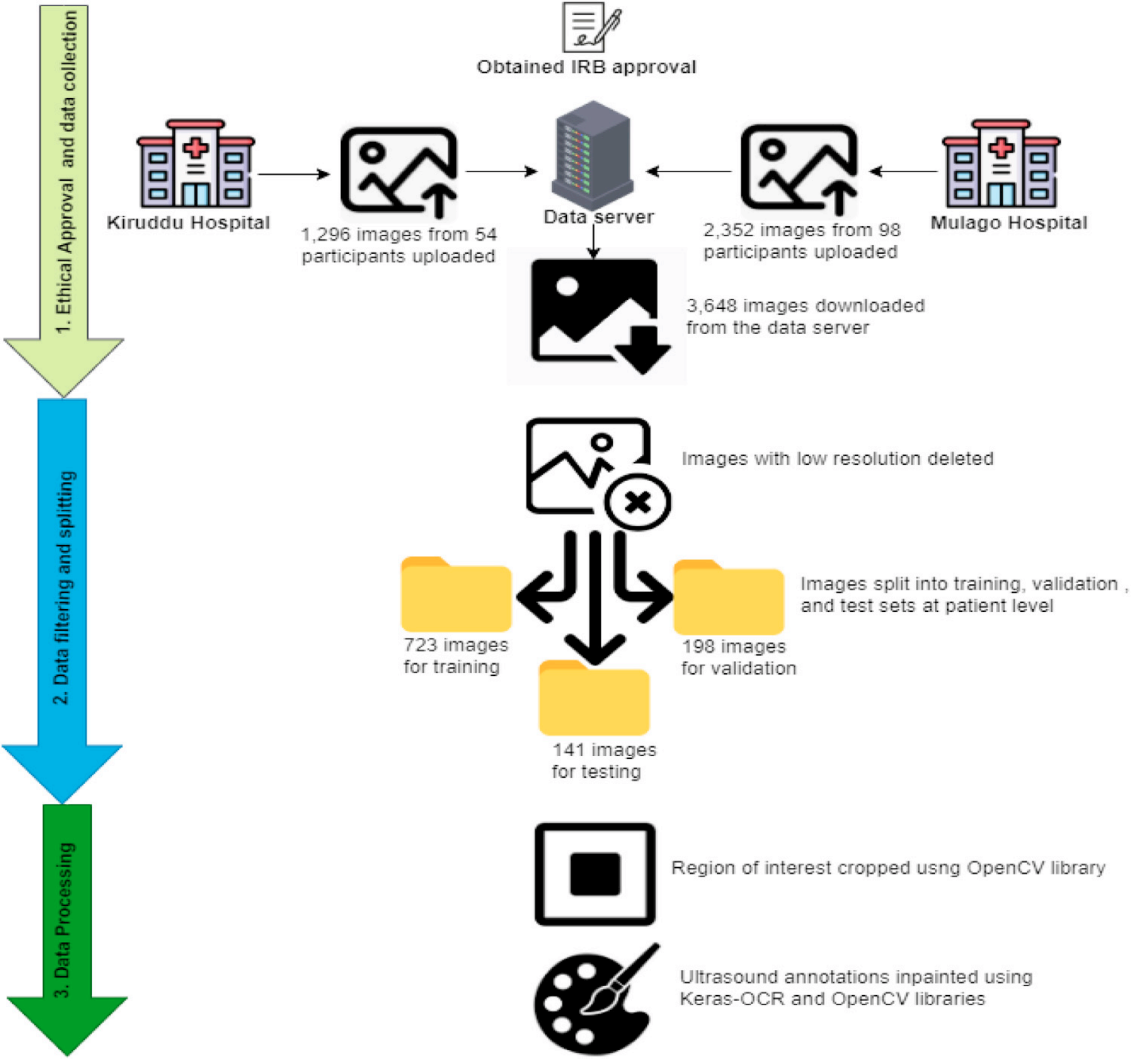
Workflow outlining the process from ethical approval and data acquisition to data filtering, sorting, and subsequent processing.

In this section, we present the workflow used to generate the dataset. Fig. 2 illustrates the complete process, from data collection to processing.

### 4.1 Ethical approval and data acquisition

Ethical clearance for this study was granted by the Mulago Hospital Research and Ethics Committee (MHREC; Approval No MHREC 2021–45, dated 24 August 2021) and the Uganda National Council for Science and Technology (UNCST; Approval No SIR74ES, dated 22 October 2021) [11]. The study enrolled participants presenting with suspected COVID-19 infection or other pulmonary pathologies, including non-COVID-19-related pneumonia. Prior to participation, written informed consent was obtained from all subjects after providing detailed information regarding the study’s objectives, data collection protocols, and participants’ rights.

Lung ultrasound data were acquired by three senior radiologists; two based at Mulago National Referral Hospital and one at Kiruddu Referral Hospital, Uganda. Each patient’s thorax was anatomically divided into 12 regions: six zones on the left to represent upper posterior, lower posterior, upper anterior, lower anterior, upper lateral, and lower lateral and six corresponding zones on the right [11]. Each region was scanned in both longitudinal and transverse orientations using Clarius C3 and C5 ultrasound probes [7], configured with the following settings: frame rate of 20 Hz, imaging depth of 16.0 cm, gain of 53, and power output of −0.3 dB. A total of 24 image frames per participant were acquired and subsequently uploaded to a secure data server for further processing.

### 4.2 Data filtering and splitting

A quality assessment of the collected images was conducted to identify and exclude low-resolution images. In consultation with radiologists, suboptimal images were removed from the dataset to ensure diagnostic relevance and enhance model robustness [11]. The remaining images were then stratified into training, validation, and test sets in a 70:20:10 ratio using a Python script. Splitting was performed based on folder identifiers to guarantee that all images from a single patient were allocated to only one subset, thereby preventing data leakage across splits.

### 4.3 Data processing

The raw ultrasound image frames contained large non-informative regions and overlaid labels, both of which posed challenges for efficient model training and accurate feature extraction. To mitigate these, challenges, a customized pre-processing pipeline was developed and implemented using OpenCV(v4.11.0), NumPy(v1.26.4),and Keras-OCR (v0.9.3) [11].

Initially, images were converted to grayscale using the OpenCV library [8], Binarization was then performed using Otsu’s thresholding method [9], which automatically selected an optimal global threshold level for foreground-background separation. The initial threshold value was set to 0 and the maximum to 255. To enhance the continuity of anatomical structures, morphological closing and dilation with a rectangular structuring element of size 2 × 2 were applied. Contour detection was subsequently carried out, and the largest contour, presumed to correspond to the lung region, was enclosed within a bounding box, allowing the image to be cropped to the region of interest.

**Fig. 3:**
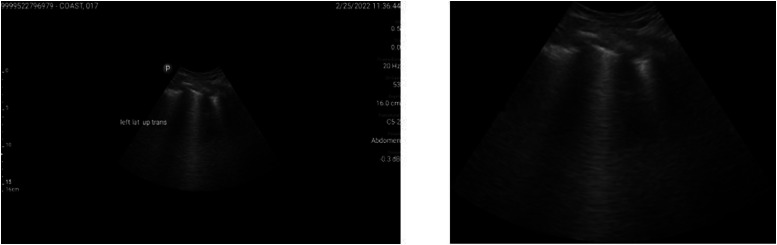
**Left:** Original image frame with non-informative regions and overlaid labels. **Right:** Corresponding processed image [11].

To remove overlaid ultrasound labels, the Keras-OCR library was employed to detect text regions [10], which were represented as quadrilaterals bounding polygons. The vertical midpoints of these quadrilaterals were used to guide the placement of linear masks, with line thickness determined proportionally based on the inter-point distances of the detected text boundaries. Inpainting was then applied to reconstruct the occluded regions by interpolating surrounding pixel values, effectively removing textual artifacts while preserving anatomical integrity. All processing steps were executed in a Google Colab environment equipped with a T4 GPU. Fig. 3 shows a representative example of the original and processed image.

## Limitations

The lung disease classification dataset consists of only three classes: COVID-19, Other lung disease, and Healthy lung. This classification was selected because the clinical study primarily aimed to identify COVID-19, leading the radiologist to focus mainly on this condition while placing less emphasis on other findings.

## Ethics Statement

The authors affirm that all human participant procedures received ethical approval from the Mulago Hospital Research and Ethics Committee (MHREC), protocol number MHREC 2021–45 and the Uganda National Council for Science and Technology (UNCST) with registration number SIR74ES. MHREC granted approval on August 24, 2021, and UNCST on October 22, 2021. Informed consent was obtained from all patients before data collection. To ensure patient privacy, the dataset was anonymized, with no identifiable information included.

## Credit Author Statement

**Andrew Katumba:** Conceptualization, Review and editing, Validation, Supervision, Project administration, Methodology, Investigation, Funding acquisition, and Formal analysis. **Sudi Murindanyi:** Conceptualization, Writing original draft, Validation, Resources, Methodology, and Formal analysis. **Nixson Okila:** Conceptualization, Writing original draft, Resources, Methodology, and Formal analysis. **Joyce Nakatumba-Nabende:** Conceptualization, Review and editing, Validation, Supervision, Project administration, Methodology, Investigation, Funding acquisition, and Formal analysis. **Cosmas Mwikirize:** Conceptualization, Review and editing, Validation, Supervision, Project administration, Methodology, Investigation, Funding acquisition, and Formal analysis. **Jonathan Serugunda:** Review and editing, Supervision, Investigation, and Funding acquisition. **Samuel Bugeza:** Data acquisition, and Data curation. **Antony Oriekot:** Data acquisition and Data curation. **Juliet Bossa:** Data curation. **Eva Nabawanuka:** Writing – review and editing, Validation, Supervision, Methodology, Investigation, Funding acquisition, Data acquisition, and Data curation.

## Data Availability

Mendeley DataA Dataset of Lung Ultrasound Images for Automated AI-based Lung Disease Classification (Original data).
